# Exclusive Enteral Nutrition Orchestrates Immunological Balances as Early as Week 4 in Adult Patients of Crohn’s Disease: A Pilot, Open-Lable Study

**DOI:** 10.3390/nu15245091

**Published:** 2023-12-13

**Authors:** Na Diao, Xinyu Liu, Minzhi Lin, Qingfan Yang, Bingyang Li, Jian Tang, Ni Ding, Xiang Gao, Kang Chao

**Affiliations:** 1 Department of Gastroenterology, The Sixth Affiliated Hospital, Sun Yat-sen University, Guangzhou 510630, China; diaon@mail.sysu.edu.cn (N.D.); liuxy529@mail2.sysu.edu.cn (X.L.); linmzh23@mail2.sysu.edu.cn (M.L.); yangqf3@mail.sysu.edu.cn (Q.Y.); 19860019675@163.com (B.L.); tangj33@mail.sysu.edu.cn (J.T.); dingn8@mail.sysu.edu.cn (N.D.); 2 Guangdong Provincial Key Laboratory of Colorectal and Pelvic Floor Diseases, The Sixth Affiliated Hospital, Sun Yat-sen University, Guangzhou 510630, China; 3 Biomedical Innovation Center, The Sixth Affiliated Hospital, Sun Yat-sen University, Guangzhou 510630, China

**Keywords:** Crohn’s disease, exclusive enteral nutrition, amino-acid-rich elemental diet, clinical remission, immune modulation, immune subsets

## Abstract

Background and aims: The efficacy and underlying mechanisms of exclusive enteral nutrition (EEN) in adult patients with Crohn’s disease (CD) remain controversial. This study aimed to evaluate the role of EEN in adult patients with CD and to explore the mechanisms from the perspective of immunoregulation. Methods: This is a prospective, open-label pilot study. Active patients with CD were enrolled and prescribed an amino-acid-rich elemental diet for 12 weeks. Dynamic changes in immune cells, including neutrophils, monocytes, T cells and B cells, were detected by flow cytometry. Plasma cytokines were evaluated by ELISA. Results: Twenty adult patients with CD were enrolled. Among them, 1 discontinued treatment due to poor compliance, and 19 patients were included for final analysis. Clinical remission was achieved in 47.37% (9/19), 63.16% (12/19), and 73.68% (14/19) patients at weeks 4, 8, and 12, respectively. Endoscopic remission and transmural healing were achieved in 52.63% (10/19) and 15.79% (3/19) patients at week 12. Notably, there was no significant difference in clinical remission between week 4 and week 8 (*p* = 0.33) or week 12 (*p* = 0.09). Furthermore, we observed a rapid reconstitution of immunologic homeostasis as early as week 4. At week 4, both the frequency and activation of neutrophils and monocytes were decreased after EEN therapy. Significant decreases in Th17 cells and naïve B cells, increases in memory B cells, and regulatory B cells were also detected. These changes remained stable at weeks 8 and 12. Conclusions: EEN with an amino-acid-rich elemental diet orchestrated immunological balances and induces clinical remission in adult CD patients as early as week 4, suggesting a 4-week EEN therapy may be feasible and practicable in clinical practice.

## 1. Introduction

Crohn’s disease (CD), a form of inflammatory bowel disease (IBD), manifests as recurrent gastrointestinal transmural inflammation and remains incurable. The exact cause of CD is unclear but has been attributed to a complex interplay among genetic risk, environmental exposures, the immune system, and the gut microbiota [1]. In clinical practice, inhibition of an abnormal immune response is the main approach to induce and maintain remission in patients with CD. However, even though new therapies were blooming in the last few decades, no one could break the ceiling of CD treatment.

Diet plays an important and paradoxical role in the progression of CD [2,3]. Dietary elements, such as simple sugars, acted as inflammation triggers [4,5], whereas other elements, such as polysaccharides, acted as anti-inflammatory agents in a colitis mouse model [6]. Strict regulation of dietary intake has been shown to help induce or maintain remission in CD. Among all emerging dietary patterns, exclusive enteral nutrition (EEN) is the most effective for resolving CD inflammation [7]. EEN, the exclusive feeding of liquid formula over 6–8 weeks, is recommended as the first-line treatment for the induction of remission in pediatric patients with CD, with approximately 80% of patients achieving remission [8]. However, the role of EEN in adult patients with CD remains less conclusive and controversial, as the response rate to EEN in adults is extremely heterogeneous, ranging from 50 to 80% [9,10,11]. In addition, due to its poor palatability and unsustainability, the acceptability of EEN was limited in adult patients with CD, especially for long-term usage. 

The specific mechanism of EEN for treating IBD is still unclear. Immune disorders mediated by innate and adaptive immune cells, including neutrophils, monocytes, T cells, and B cells, play a critical role in the progression of IBD. Feeding with EEN reduces interleukin 17α (IL-17α) and vascular endothelial growth factor (VEGF) and increases transforming growth factor (TGFβ) expression [12,13]. Moreover, EEN was shown to increase FOXP3^+^ regulatory T cell expansion and then correct the pro- and anti-inflammatory imbalances to promote inflammation resolution [14]. However, the immune regulatory function of EEN remains largely unknown. A better understanding of how EEN works will help promote its use in the treatment of patients with CD.

According to the protein composition, the enteral formulas used in patients with CD mainly include two types: polymeric formulas and elemental formulas that are comprised of peptides and amino-acid-enriched formulas. Among them, polymeric-based enteral formulas are most widely used in patients with CD. However, based on previous studies, differences in nitrogen sources in enteral formulas were not relevant to their therapeutic efficacy, as polymeric and elemental diets were equally effective [15,16].

In the present study, we performed a prospective, open-label pilot study to assess the efficiency of EEN with an amino-acid-rich elemental diet in adult patients at weeks 4, 8, and 12 and to examine the immune-modulatory function of EEN in human subjects.

## 2. Materials and Methods

### 2.1. Patients and Methods

This study was approved by the Medical Ethical Committee of the Sixth Affiliated Hospital (2022ZSLYEC-019) and registered in the Chinese Clinical Trial (ChiCTR1900026677). Consecutive patients with CD who visited our IBD center between 1 December 2019 and 30 November 2020 were included in the study according to the inclusion and exclusion criteria. In accordance with this approval, patients provided informed consent for the use of their samples in this study. The diagnosis of CD was based on the current guidelines, including clinical, radiographic, endoscopic, and histological findings [17]. Disease location, age of disease onset, and disease behavior were classified using the Montreal classification. Inclusion criteria were patients with (1) confirmed diagnosis of CD, (2) Crohn’s Disease Activity Index (CDAI) > 150, (3) aged from 18 to 55 years, and (4) signed informed consent. Exclusion criteria were patients (1) who received antibiotics or probiotics within 2 weeks; (2) who were prescribed corticosteroids, biological agents, or immunomodulatory drugs within 1 month; (3) who were administered concomitant medications; or (4) who were previously diagnosed with short bowel syndrome, high output fistula, complete intestinal obstruction, cancer, or autoimmune disease.

Patients enrolled in this study were fed liquid formula, Elental^®^ (Ajinomoto and Morishita-Russel, Tokyo, Japan), by a nasogastric tube for 12 weeks. Elental^®^ is an elemental diet that is composed of amino acids, low amounts of fat, vitamins, trace elements, and dextrin as a major energy source (Supplementary Table S1) with a calorie density of 1 kcal/mL and an osmolarity of 760 mOsm/L.

Demographic data were collected at baseline. Patients were followed up and underwent full assessments prior to and after EEN therapy at weeks 0, 4, 8, and 12. At weeks 0, 4, 8, and 12, body mass index (BMI), CDAI, clinical parameters [high-sensitivity C-reactive protein (hs-CRP) and erythrocyte sedimentation rate (ESR)], cytokine levels, serum albumin (ALB), and hemoglobin (Hb) were assessed and collected. At weeks 0 and 12, endoscopy, computed tomography enterography (CTE), and magnetic resonance imaging (MRI) of the anal canal were performed. 

### 2.2. Outcomes and Definitions

The primary outcome was clinical remission at weeks 4, 8, and 12. The secondary outcomes were endoscopic remission and transmural healing at week 12. CDAI > 150 was considered an active disease. CDAI ≤ 150 was considered clinical remission. The Crohn’s Disease Endoscopic Index of Severity (CDEIS) was determined based on the endoscopic findings [17,18]. CDEIS < 3 or a drop of >70% was considered as endoscopic remission. The findings of CTE, including abnormal mucosal/mural enhancement, mesenteric hypervascularity, mesenteric inflammatory fat stranding, and bowel wall thickening, were scored [18,19]. Transmural healing was defined as the normalization of bowel wall thickness (≤3 mm) and absence of the lesions, including mural hyperenhancement, increased mesenteric adipose tissue concentration, and fistula or abscess, of all inflamed segments involved in CD on CTE [19,20,21].

### 2.3. Flow Cytometry

The distribution and function of neutrophils, monocytes, T cells, and B cells were assessed and analyzed by flow cytometry. Peripheral blood samples were obtained from 15 patients undergoing EEN treatment at weeks 0, 4, 8, and 12. Additionally, for the analysis of immune subsets, fourteen healthy sex and age-matched adults were recruited as a control group and were not given EEN over the duration of the study. Peripheral blood cells were depleted with red blood cells and stained with anti-CD14, anti-CD15, anti-CD16, anti-CD54 (ICAM-1), anti-CD4, anti-CCR4, anti-CCR6, anti-CXCR5, anti-CD19, anti-CD24, anti-CD27, and anti-CD38 antibodies (BioLegend, San Diego, CA, USA). The samples were then analyzed using a FACSCanto II (BD Biosciences, San Jose, CA, USA). The data were processed by Flow Jo (Tree Star, Ashland, OR, USA).

### 2.4. Plasma Cytokine Assays and Blood Tests

At weeks 0, 4, 8, and 12, 3 mL of peripheral blood was obtained from patients with CD treated with EEN, and plasma samples were obtained by centrifugation. Cytokine levels were examined by enzyme-linked immunosorbent assay (ELISA) according to the manufacturer’s instructions (eBioscience, San Diego, CA, USA).

Serum ALB (reference range 40–55 g/L) was determined by the bromocresol green method (Beckman Coulter, Brea, CA, USA). Hs-CRP (0–10 mg/mL) was quantitatively measured with a rate immune scatter turbidimetry method (Beckman Coulter). Red blood cell (RBC, 4.0–5.5 × 10^12^ cells/L) and platelet (PLT, 100–300 × 10^9^ cells/L) counts were analyzed using electrical impedance assay (Mindray, Shenzhen, China). Hb (120–160 g/L for males, 110–150 g/L for females) in whole blood was quantitatively measured using the Hemoglobin Colorimetric Detection Kit (Mindray, China). ESR (0–38.4 mm/h) were measured by the Westergren method (Mindray, China).

### 2.5. Statistical Analysis

The expected clinical remission rate of EEN therapy with amino-acid-based formulas was 80%, according to clinical experience and a prior report [11]. Setting an acceptable 95% confidence interval width (two-sided) of 30%, the calculated sample size was 26. Assuming a dropout rate of 10%, 29 patients were expected to be included. Data are expressed as the mean ± SEM or the median and range. Statistical significance between groups was determined using one-way analysis of variance (ANOVA) with Tukey’s multiple comparison test. Differences between the two groups were evaluated using paired or unpaired two-tailed Student’s t tests, and *p* values ≤ 0.05 were considered statistically significant. All statistical analyses were performed using SPSS software, ver. 19.0. 

## 3. Results

### 3.1. Demographic and Clinical Characteristics of the Study Population

Twenty patients were prospectively included, and 1 patient withdrew from the study one week after the initiation of EEN therapy due to poor compliance. Eventually, 19 patients were successfully included for further analysis, including 12 males (63.16%) and 7 females (36.84%). The median age at study inclusion was 25 years (range 18–40 years). Among them, 18 patients (94.74%) had moderate and severe CD with CDAI > 220. Non-stricturing non-penetrating disease (B1), stricturing disease (B2), and penetrating disease (B3) were seen in 31.58%, 31.58%, and 36.84% of patients at baseline, respectively. Seven patients were complicated with an intestinal fistula, of which 5 patients (5/7) had non-drainable abdominal abscesses. Additionally, 17 cases (89.47%) were complicated with perianal fistulas. The demographic data at baseline are shown in Table 1.

### 3.2. EEN Effectively Induces Clinical, Endoscopic, and Transmural Remission in Adults with CD

**Figure 1:**
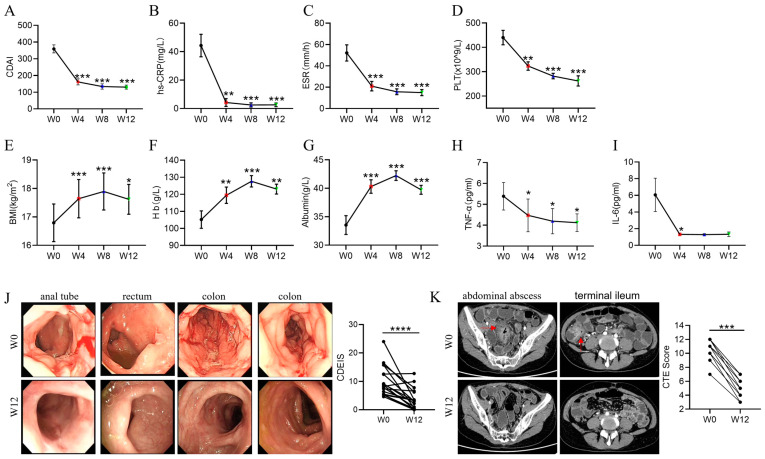
The dynamic changes in clinical parameters in adult patients with CD during EEN treatment. (**A**–**D**) Changes in CDAI (**A**), hs-CRP (**B**), ESR (**C**), and number of platelets (**D**) in adult patients with CD during EEN treatment. (**E**–**G**) Changes in BMI (**E**), hemoglobin (**F**), and albumin (**G**) in adult patients with CD during EEN treatment. (**H**,**I**). Dynamic changes in IL-10/TNF-α (**H**) and IL-10/IL-6 (**I**) in patients with CD during EEN therapy. (**J**) Images of colonoscopy (**left**) of representative patient with CD at weeks 0 and 12 after EEN therapy. Statistical analysis (**right**) of CDEIS of patients with CD before and after EEN therapy. (**K**) Images of CTE (**left**) of representative patient and CTE score (**right**) of patients with CD at weeks 0 and 12 after EEN therapy. The red arrows indicated ileocolonic lesions. * *p* < 0.05, ** *p* < 0.01, *** *p* < 0.001, **** *p* < 0.0001.

After the initiation of EEN therapy, clinical response was observed in 94.74% (18/19), 100.00% (19/19), and 100.00% (19/19) patients at weeks 4, 8, and 12, respectively. Clinical remission was noted in 47.37% (9/19), 63.16% (12/19), and 73.68% (14/19) of patients at weeks 4, 8, and 12, respectively. No significant differences in the clinical remission rate were noted between week 4 and week 8 (*p* = 0.33) or week 12 (*p* = 0.09). The CDAI of patients was 354.75 ± 110.72 at inclusion and decreased significantly over time, with 168.68 ± 67.07 (*p* < 0.001), 138.41 ± 64.89 (*p* < 0.001), and 130.8 ± 50.91 (*p* < 0.001) at weeks 4, 8, and 12, respectively (Figure 1A). Meanwhile, clinical inflammatory parameters, including hs-CRP, ESR, and PLT, were significantly reduced at week 4 compared with week 0 (*p* < 0.001, *p* < 0.001, *p* = 0.002, respectively) (Figure 1B–D). Whereas other parameters, such as BMI, Hb, and ALB, were significantly increased at week 4 compared with week 0 (*p* < 0.001, *p* = 0.048, *p* < 0.001, respectively) (Figure 1F,G). All these changes remained stable at weeks 8 and 12. Consistently, both TNF-α and IL-6 decreased significantly at week 4 (*p* = 0.02, *p* = 0.048) after EEN treatment and remained at a low level until week 12 (Figure 1H,I).

In addition, the rate of endoscopic remission was up to 52.63% (10/19) at week 12. The CDEIS was reduced from 10.3 ± 5.21 at week 0 to 3.98 ± 3.63 at week 12 (*p* < 0.001) (Figure 1K). Furthermore, the CTE score was reduced from 10.4 ± 1.36 at week 0 to 5.2 ± 1.31 at week 12 (*p* < 0.001) (Figure 1J). And 15.79% (3/19) of patients achieved transmural healing at week 12. In detail, all 3 patients with transmural healing were B1-type (50%, 3/6). For 7 patients complicated with intestinal fistulas, fistula closure was observed in 4 patients and fistula improvement in 3 patients at week 12. For 5 patients with abdominal abscesses, the abscesses disappeared in 3 patients and obviously diminished in 2 patients at week 12. Taken together, our data showed that EEN was an effective method to induce rapid clinical, endoscopic, and transmural remission and improve complications in adults with CD.

### 3.3. Reduced Neutrophil Accumulation and Activation in Patients with CD Treated with EEN

**Figure 2:**
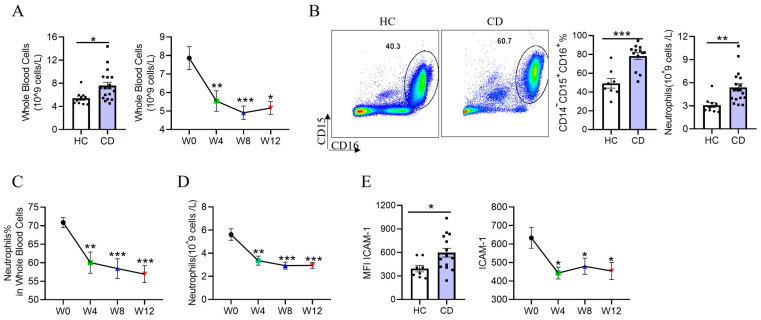
Alterations in neutrophil distribution and function in healthy controls and adult patients with CD during EEN treatment. (**A**) The absolute number of whole blood cells from healthy controls and adult patients with active CD administered EEN therapy. (**B**) Frequency of neutrophils (CD15^+^CD16^+^) in peripheral blood from healthy controls and adult patients with active CD. (**C**,**D**) Changes in the frequency (**C**) and number (**D**) of neutrophils in peripheral blood from adult patients with active CD before and after EEN treatment. (**E**) Changes in ICAM-1 expression on neutrophils obtained from healthy controls and adult patients with active CD during EEN therapy. * *p* < 0.05, ** *p* < 0.01, *** *p* < 0.001.

For the analysis of immune subsets, fourteen healthy controls were recruited as a control group. No significant differences in age and sex distribution at the initial diagnosis were noted between patients and controls. Previous studies reported that the number of neutrophils was positively correlated with the severity of IBD [21,22]. ICAM-1, as one of the key molecules of neutrophil activation, was increased in the intestinal mucosal tissues of IBD, promoting inflammation by mediating the aggregation, migration, and activation of neutrophils [23]. Hence, we analyzed whether neutrophils and their surface functional molecule ICAM-1 mediate EEN-induced inflammatory remission in CD. Firstly, we discovered that the absolute number of white blood cells and neutrophils, the percentage of neutrophils, and ICAM-1 expression on neutrophils were significantly upregulated in patients with active CD compared with healthy controls (Figure 2A,B,E). During the course of EEN therapy, the absolute number of whole blood cells, the absolute number and percentage of neutrophils, and ICAM-1 expression on neutrophils were reduced significantly at week 4 and remained at low levels until the end of the experiment at week 12 (Figure 2A,C–E). Collectively, neutrophil activation and accumulation were dramatically suppressed by EEN as early as week 4.

### 3.4. Decreased Frequency of Inflammatory Monocytes in Patients with CD Treated with EEN

**Figure 3:**
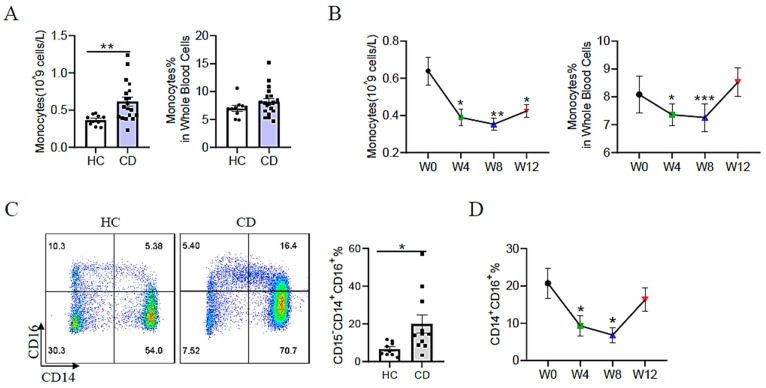
The changes in monocyte subsets in healthy controls and adult patients with CD during EEN treatment. (**A**) The absolute number and frequency of monocytes in peripheral blood obtained from healthy controls and adult patients with active CD. (**B**) Changes in the number and frequency of neutrophils in peripheral blood from adult patients with active CD before and after EEN treatment. (**C**) Frequency of CD14^+^CD16^+^ monocytes in peripheral blood obtained from healthy controls and adult patients with active CD. (**D**) Change in the frequency of CD14^+^CD16^+^ monocytes in peripheral blood from patients with CD during EEN therapy. * *p* < 0.05, ** *p* < 0.01, *** *p* < 0.001.

Proinflammatory CD14^+^CD16^+^ monocytes promote inflammatory progression in IBD [22]. In the present cohort, the number of total monocytes and the frequency of CD14^+^CD16^+^ monocytes significantly increased in patients with active CD compared with healthy controls at study initiation (Figure 3A,C). During the course of EEN therapy, the absolute number of monocytes was reduced significantly at week 4 and remained at low levels at weeks 8 and 12 (Figure 3B). The frequency of monocytes in blood cells and the frequency of proinflammatory monocytes (CD14^+^CD16^+^) in monocytes were reduced at weeks 4 and 8 and increased to approximately the initial level at week 12 (Figure 3B,D). These data indicated the expansion of circulating proinflammatory monocytes was inhibited by EEN at the early stages of weeks 4 and 8.

### 3.5. EEN Inhibits Th17 Cell Differentiation in Adult Patients with Active CD

**Figure 4:**
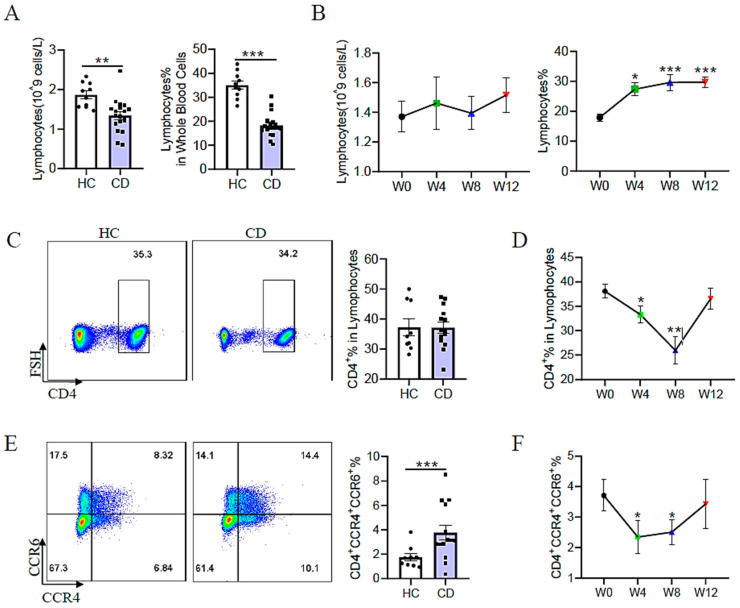
Variations in distinct T helper cells in healthy controls and adult patients with CD during EEN treatment. (**A**) The absolute number and frequency of lymphocytes from healthy controls and adult patients with active CD. (**B**) The number and frequency change in lymphocytes in peripheral blood from patients with CD during EEN therapy. (**C**) The frequency of circulating CD4^+^ T cells in healthy controls and adult patients with active CD. (**D**) Changes in the frequency of circulating CD4^+^ T cells from patients with CD during EEN therapy. (**E**) The frequency of circulating Th17 cells (CD4^+^CCR4^+^CCR6^+^) in healthy controls and adult patients with active CD. (**F**) Changes in the frequency of circulating Th17 cells in patients with CD during EEN therapy. * *p* < 0.05, ** *p* < 0.01, *** *p* < 0.001.

In addition to innate immune cells, the adaptive immune response represented by T helper cells plays a more fundamental role in the progression of IBD. In our study, we mainly focused on the role of EEN in Th17 cells. Before the initiation of EEN therapy, flow cytometry revealed that the number and percentage of lymphocytes were significantly decreased compared to those in healthy controls (Figure 4A). No significant difference in the frequency of total CD4^+^ T cells was noted between patients with active CD and healthy controls (Figure 4C). Whereas, an increased frequency of Th17 (CD4^+^CCR4^+^CCR6^+^) cells was noted in adult patients with active CD compared with healthy controls (Figure 4E). During the course of EEN therapy, we observed an increase in the percentage of lymphocytes in white blood cells at week 4 and remained at a high level until week 12 (Figure 4B), while the absolute number of lymphocytes was not altered during the period of EEN treatment (Figure 4B). Meanwhile, the percentage of CD4^+^ T cells in lymphocytes and the percentage of Th17 cells in CD4^+^ T cells were reduced at weeks 4 and 8 and increased approximately back to the initial level at week 12 (Figure 4F). 

### 3.6. EEN Helps to Modify the B-Cell Immune Response

**Figure 5:**
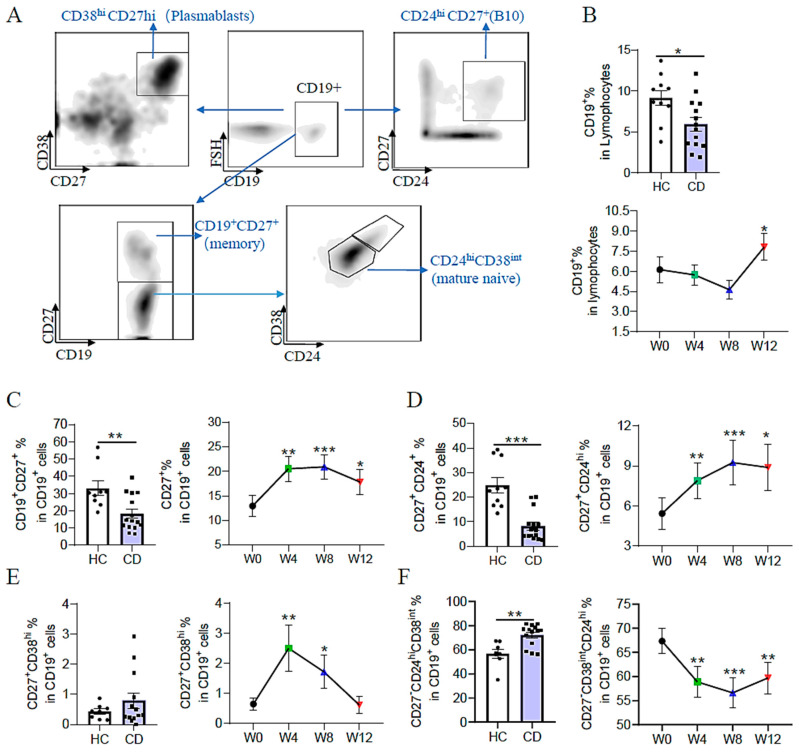
B-cell immune response in healthy controls and adult patients with CD. (**A**) Gating strategy of B-cell subsets. (**B**) The frequency of circulating B cells (CD19^+^) in healthy controls and adult patients with active CD, and in patients with CD during EEN therapy. (**C**) The frequency of memory B cells (CD27^+^) in healthy controls and adult patients with active CD, and in patients with CD during EEN therapy. (**D**) The frequency of CD27^+^CD24^hi^ B cells in healthy controls and adult patients with active CD, and in patients with CD during EEN therapy. (**E**) The frequency of CD27^+^CD38^hi^ plasmablast B cells in healthy controls and adult patients with active CD, and in patients with CD during EEN therapy. (**F**) The frequency of CD27^−^CD38^int^CD24^hi^ mature naive B cells in healthy controls and adult patients with active CD, and in patients with CD during EEN therapy. * *p* < 0.05, ** *p* < 0.01, *** *p* < 0.001.

The role of B cells in IBD and their association with EEN treatment remained largely unknown. Therefore, B-cell subsets in healthy controls and adult patients with active CD were investigated according to the gating strategy shown in Figure 5A. Compared with healthy controls, the percentage of B cells (CD19^+^) in lymphocytes was dramatically reduced in patients with CD (Figure 5B). Meanwhile, patients with CD exhibited significantly lower percentages of memory B cells (CD27^+^) and regulatory B cells (CD24^hi^CD27^+^) but higher percentages of naïve B cells (CD27^−^CD24^hi^CD38^int^) compared with healthy controls (Figure 5C,D,F). Within the antibody-producing B cell compartment, some patients were detected with slightly elevated frequencies of plasmablasts (CD27^+^CD38^hi^) (Figure 5E), whereas no significant difference in the frequency of plasmablasts was noted between patients with CD and healthy individuals. These data implied that the B-cell immune response was disrupted in adult patients with active CD.

After EEN treatment, the percentage of B cells in lymphocytes declined slightly at weeks 4 and 8, while increasing significantly at week 12 (Figure 5B). Taking a closer look at the different B-cell subsets, CD27^+^ memory B cells and CD27^+^CD24^hi^ regulatory B cells were increased significantly, whereas naïve B cells (CD27^−^CD24^hi^CD38^int^) were decreased significantly at week 4 after initiation of EEN therapy (Figure 5C,D,F). These changes remained stable until the endpoint at week 12 in the course of EEN therapy. Interestingly, we found that the proportion of plasmablasts increased significantly at week 4 after EEN treatment and decreased back to the initial level at week 12 (Figure 5E). In summary, our data exhibited that the B-cell immune response was disrupted in adult patients with active CD, and EEN therapy could help to modify the B-cell immune reaction as early as week 4.

## 4. Discussion

In the present prospective, open-label pilot study, we provided evidence about the effectiveness of EEN with an amino-acid-rich formula in adult patients with CD, not only on clinical remission but also on deep remission. Notably, we reported the dynamic changes of the immune subsets over time during the EEN treatment systemically, which may be helpful in discovering the mechanisms of EEN.

There was high-grade evidence of EEN therapy in pediatric patients with CD but heterogenous data in adult patients with CD. In our study, Elental^®^, an amino acid-based elemental formula, was prescribed to adult patients with CD by a nasogastric tube for 12 weeks. Elental^®^ has been widely used as a nutritional supplement for CD patients [24,25].The clinical remission was reported to be 71% after 4 weeks of enteral nutrition with Elental^®^, and endoscopic healing rates were 39% and 44% in the large bowel and terminal ileum, respectively [24]. In the present study, the clinical remission rate of EEN with Elental^®^ in adult CD was 47.37%, 63.16%, and 73.68% at weeks 4, 8, and 12, respectively. Additionally, 52.63% of patients prescribed EEN reached endoscopic remission at week 12. Meanwhile, EEN treatment also reduced inflammatory cytokines such as hs-CRP, ESR, PLT, and TNFα. The results were in line with previous reports, which indicated that EEN with Elental^®^ is effective for inducing clinical and endoscopic remission as well as reducing inflammatory makers in adult CD [24,25]. Enteral nutritional (EN) formulas include polymeric formulas and elemental formulas based on protein composition. Previous studies showed there were no significant differences between elemental formula and polymeric formula on clinical remission rate at week 4 in adult CD (80% vs. 55%, *p* = 0.1) and at week 6 in pediatric CD (69% vs. 82%, *p* = 0.44) [15,26]. A meta-analysis also confirmed that formula composition did not influence the efficacy of EEN [27].Thus, the results of this study of EEN with an amino-acid-rich formula may reflect the efficacy of EEN in adult patients with CD. Therefore, we also compared the results with those of other studies with different formulas. Consistent with the present study, the meta-analysis has clearly shown the effectiveness of enteral nutrition in inducing clinical remission in adult patients with CD [27]. Transmural healing is an emerging treatment target for CD. A few studies reported a transmural healing rate of 17% and 21% with polymeric-based enteral nutrition in adult or pediatric CD [28]. In the present study, the rate was similar (15.79%). Notably, more than 30% of patients have penetrating diseases, which is a risk for a poor prognosis. Indeed, all the patients who achieved transmural healing in short-term EEN therapy had a B1 phenotype. However, the patients with fistulas or abscesses also benefited from EEN. 

According to the current guideline [29], corticosteroids, biologicals, and JAK inhibitors can be used to induce remission in adult patients with CD. A few studies and meta-analyses have compared the efficacy of corticosteroids and EEN. Very low-quality evidence suggests that corticosteroid therapy may be more effective than EN for the induction of clinical remission in adults with active CD. Evidence also suggests that EN may be more effective than steroids for the induction of remission in children with active CD [27]. In another meta-analysis, there was no difference in efficacy between EEN and corticosteroids in induction remission (OR = 1.26 [95% CI 0.77, 2.05]) in Crohn’s disease in a pediatric population [30]. It was confusing about the different results between adult and pediatric patients. It was shown that patients on EN were more likely to withdraw due to adverse events than those on steroid therapy, especially in adult patients, which may be the cause of the difference [27]. For anti-TNF therapy, remission rates were highly variable across the different studies, with short-term rates between 41% and 83% [31]. A previous study demonstrated that EEN was comparable to anti-TNF at week 8 on clinical response [32]. Head-to-head studies to compare the efficacy or EEN and other drugs were limited; however, in phase 3 clinical trials of the current medications, induction remission rates of 36.3–56.8% were reported [33,34,35].The rates were similar to the present study. Moreover, data about the efficacy of medications in penetrating diseases was limited, especially in patients with abscesses. The emerging evidence supports the idea that EEN may be a good choice for both effectiveness and reduced risks of infection.

Immunological imbalances were believed to play a critical role in the pathogenesis of CD. Although biologics and small molecular drugs have emerged to treat CD, it is difficult to handle the inflammation with only one or two targets in the short term. Surprisingly, EEN could restore immune hemostasis quickly in our study, without the risk of infection or other adverse events, such as glucocorticoids. Firstly, regarding innate immune cells, it was reported that the number of neutrophils and pro-inflammatory monocytes (intermediate human CD14^+^CD16^+^) monocytes was positively correlated with the severity of IBD [22,36,37]. Activated neutrophils and pro-inflammatory monocytes that accumulate in the circulation and colon tissue could aggravate intestinal inflammation by releasing inflammatory mediators [22,36]. Our data revealed that after EEN therapy, both the frequency and activity of the neutrophils were significantly reduced from week 4 and remained at low levels until week 12 in adult patients with CD. In addition, the percentage of inflammatory monocytes (CD14^+^CD16^+^) showed the same tendency. Secondly, EEN also modulated the adaptive immune cell subsets. It was previously reported that EEN therapy increased FOXP3^+^ Tregs and reduced Th1-derived IFN-γ [14]. So, in our study, we mainly focused on the role of EEN in Th17 cells. Th17 cells mainly play a proinflammatory role in the pathogenesis of IBD and exacerbate the intestinal inflammatory response through the proinflammatory cytokines IL-17/IL-22 [38].We observed that upon EEN treatment, the frequency of Th17 cells decreased significantly at weeks 4 and 8. Thirdly, the role of the B cell subset in CD was largely unclear. It may have both pathogenic and protective functions in IBD [39]. Naïve B cells aggravated inflammation by mediating antibody production, whereas regulatory B cell subsets were believed to suppress abnormal immune responses in autoimmune disease [40]. In our study, we were able to longitudinally analyze the distribution of four distinct B-cell subsets in the adult CD cohort during the course of EEN therapy. For memory and naïve B-cell subsets, our data revealed decreased proportions of CD27^+^ memory B cells and increased proportions of naïve B cells in patients with CD, which was consistent with the current literature [41]. After the initiation of EEN therapy, the proportions of CD27^+^ memory cells increased, whereas naïve B cells decreased, indicating that EEN helped to restore the balance of CD27^+^ memory and naïve B cells. Concerning regulatory B cells, we mainly assessed CD27^+^CD24^hi^ B cells. In the course of EEN therapy, CD27^+^CD24^hi^ B cells increased from week 4 to week 12, indicating that EEN helped to promote regulatory B-cell expansion. We found that the proportion of plasmablasts increased significantly at week 4, indicating that EEN facilitates antibody production at initiation, but the clinical significance behind this still needed to be elucidated. Interestingly, all of the changes could be observed as early as week 4, suggesting EEN could restore the immune balances quickly.

As we documented above, even though EEN is an effective method for inducing remission in adult CD, there are still lots of limitations to its usage in clinical practice due to diet restriction, inconvenience, and unsustainability. The efficiency of EEN is also controversial, and evidence is insufficient in adult CD, which may partially be caused by poor tolerance and compliance [42]. In this cohort, only one patient quit therapy because of poor compliance. However, the patients were more likely to follow the treatment after signing the consent form and receiving free formula. In the real-world setting, a relatively lower proportion of patients could accept 12-week EEN therapy. Shortening the treatment course for EEN may improve its acceptance. Notably, in this study, we found some clues to shorten the course to 4 weeks with both clinical and immunological data. In this study, it is surprising that 94.74% of patients received a response, and about a half of patients reached clinical remission as early as week 4. The immunological imbalances could also be restored as early as week 4, which was consistent with the clinical efficacy. These data suggest that EEN for 4 weeks may be a feasible and practicable choice for patients with active and complicated CD.

Our present study reported the clinical efficacy of EEN in adult patients as well as the underlying immunological mechanisms. It could be directly related to the elemental diet or through the complex crosstalk of diet-host-microbiota interactions. However, in a strictly designed study excluding most of the confounders, we have shown that the key driving factor of these changes was EEN therapy. The underlying crosstalk needs to be clarified in further study. There were several limitations in this study. Firstly, the sample size was small. Due to the extremely strict inclusion criteria, only 20 patients were enrolled, which was less than the estimated sample size. Although a post-hoc analysis showed higher than 80% statistical power can be achieved in the current sample size, larger cohorts are needed to confirm the results and conduct subgroup analysis. Secondly, the comparison was made on clinical variables before and after EEN intervention without patients without EEN as controls. Data on the comparative effectiveness of EEN and other medications are needed to explore this.

## 5. Conclusions

In conclusion, this study comprehensively evaluates the clinical efficacy of EEN with an amino-acid-rich formula in adult CD patients at different timepoints, incorporating dynamic analysis of innate and adaptive immune responses. The results highlighted that EEN could quickly and effectively induce clinical, endoscopic, and transmural remission and improve complications in adults with CD. The efficacy of EEN may be associated with its immune regulatory effects. Considering the dynamic changes in clinical efficacy and immunological status in this study, a 4-week EEN strategy to induce CD remission may be practical in a real-world setting.

## Figures and Tables

**Table 1:** Baseline characteristics of enrolled patients with CD.

	(*n* = 19)
Mean age, y (±SD)	26.42 ± 5.36
Gender (male, %)	63.16 (12/19)
Disease duration, y (±SD)	2.95 ± 2.32
Mean CDAI (±SD)	354.75 ± 110.72
Mean CDEIS (±SD)	10.3 ± 5.21
Mean CTE score (±SD)	10.44 ± 1.36
Age (%)	
A1	5.26 (1/19)
A2	94.74 (18/19)
A3	0
Disease location (%)	
L1	0
L2	0
L3	100
Disease Behavior (%)	
B1	31.58 (6/19)
B2	31.58 (6/19)
B3	36.84 (7/19)
P	89.47 (17/19)
Intestinal fistula (%)	36.84 (7/19)
Abdominal abscess (%)	26.32 (5/19)

Montreal classification of Crohn’s disease (CD); Age at diagnosis (A): A1 below 16 years old, A2 between 17 and 40 years old, A3 above 40 years old; Disease location (L): L1 terminal ileum, L2 colon, L3 ileocolonic, L4 upper gastrointestinal tract; Disease behavior (B): B1 non-stricturing non-penetrating; B2 stricturing, B3 penetrating, P perianal disease. CDAI, Crohn’s Disease Activity Index; CDEIS, Crohn’s Disease Endoscopic Index of Severity; CTE, computed tomography enterography; SD, standard deviation.

## Data Availability

Data are contained within the article.

## Supplementary Materials

The following supporting information can be downloaded at: https://www.mdpi.com/article/10.3390/nu15245091/s1, Table S1: Composition Amount of Elental^®^ per package.
