# Supplementary material for: Exclusive Enteral Nutrition Orchestrates Immunological Balances as Early as Week 4 in Adult Patients of Crohn’s Disease: A Pilot, Open-Lable Study
Source: Nutrients. 2023 Dec 13;15(24):5091. doi: 10.3390/nu15245091 (PMC10745958; doi:10.3390/nu15245091)
Supplement: Supplementary file 1 [file nutrients-15-05091-s001.zip › nutrients-2690053-supplementary.pdf]

nutrients

MDPI

Supplementary Materials

Exclusive Enteral Nutrition Orchestrates Immunological Balances as Early as Week 4 in Adult Patients of Crohn's Disease: A Poilt, Open-lable Study

Na Diao [1,2,3,†], Xinyu Liu [1,2,3,†], Minzhi Lin [1,2,3], Qingfan Yang [1,2,3], Bingyang Li [1,2,3], Jian Tang [1,2,3], Ni Ding [1,2,3], Xiang Gao [1,2,3,*] and Kang Chao [1,2,3,*]

Table S1. Composition Amount of Elental® per package.

Total	80g (300 kcal)	Amino acids	14.08g
Amino acids	14.08	L-Isoleucine	642.40mg
Carbohydrate(dextrin)	63.41g	L-Leucine	899.20mg
Lipid (soybean oil)	0.51g	L-Lysine-HCI	888.00mg
Vitamin A	648.00 IU	L-Methionine	648.00mg
Vitamin D	51.20 IU	L-Phenylalanine	871.20mg
Vitamin B1	0.19 mg	L-Threonine	523.20mg
Vitamin B2	0.20 mg	L-Tryptophan	151.20mg
Vitamin B6	0.26 mg	L-Valine	700.80mg
Niacin	2.22 mg	L-Histidine-HCI-H2O	500.80mg
Pantothenic acid	1.10 mg	L-Arginine-HCI	1124.80mg
Folic acid	44.00 ug	L-Alanine	899.20mg
Vitamin B12	0.70 ug	Mg-K-L-Aspartate	1036.00mg
Vitamin C	7.80 mg	Na-L-Aspartate-H2O	867.20mg
Vitamin K	9.04 ug	L-Glutamine	1932.00mg
Vitamin E	3.30 IU	Glycine	504.80mg
Biotin	38.04 ug	L-Proline	630.40mg
Choline	8.56 mg	L-Serine	1159.20mg
Na	260.00 mg	L-Tyrosine	110.40mg
K	217.60 mg		
Cl	516.80 mg		
Mg	40.00 mg		
Ca	157.60 mg		
P	121.6 mg		
Fe	1.80mg		
I	15.20ug		
Mn	300.00ug		
Cu	200.00ug		
Zn	1.80ug		

Abbreviations: Na, sodium; K, potassium; Cl, chlorine; Mg, magnesium; Ca, calcium; P, phosphorus; Fe, iron; I, iodine; Mn, manganese; Cu, copper; Zn, zinc.
